# Precision Medicine in Prostate Cancer with a Focus on Emerging Therapeutic Strategies

**DOI:** 10.3390/biomedicines14010052

**Published:** 2025-12-25

**Authors:** Ryuta Watanabe, Noriyoshi Miura, Tadahiko Kikugawa, Takashi Saika

**Affiliations:** Department of Urology, Ehime University Graduate School of Medicine, Toon 791-0295, Japan

**Keywords:** prostate cancer, precision medicine, genetic alterations, therapeutic targets, molecular subtypes

## Abstract

Precision medicine has reshaped the clinical management of prostate cancer by integrating comprehensive genomic profiling, biomarker-driven patient stratification, and the development of molecularly targeted therapeutics. Advances in next-generation sequencing have uncovered diverse genomic alterations—including homologous recombination repair defects, MSI-H/MMRd, *PTEN* loss, *BRCA1/BRCA2* mutations, *ATM* alterations, *SPOP* mutations, and molecular hallmarks of neuroendocrine differentiation—that now inform individualized treatment decisions. This review synthesizes established clinical evidence with emerging translational insights to provide an updated and forward-looking overview of precision oncology in prostate cancer. Landmark trials of PARP inhibitors and PSMA-targeted radioligand therapy have redefined treatment standards for biomarker-selected patients. Concurrently, efforts to optimize immune checkpoint inhibition, AKT pathway targeting, and rational combinations with androgen receptor pathway inhibitors continue to expand therapeutic possibilities. Rapidly evolving investigational strategies—including bipolar androgen therapy (BAT), immunotherapeutic approaches for *CDK12*-altered tumors, targeted interventions for *SPOP*-mutated cancers, and epigenetic modulation such as EZH2 inhibition for neuroendocrine prostate cancer—further illuminate mechanisms of tumor evolution, lineage plasticity, and treatment resistance. Integrating multi-omics technologies, liquid biopsy platforms, and AI-assisted imaging offers new opportunities for dynamic disease monitoring and biology-driven treatment selection. By consolidating current clinical practices with emerging experimental directions, this review provides clinicians and researchers with a comprehensive perspective on the evolving landscape of precision medicine in prostate cancer and highlights future opportunities to improve patient outcomes.

## 1. Introduction

Prostate cancer (PCa) is a common malignancy among men, with high incidence and mortality rates. In particular, advanced and treatment-resistant cases continue to carry a poor prognosis. While therapeutic options have expanded with the advent of androgen deprivation therapy (ADT) and second-generation androgen receptor (AR) inhibitors, many patients ultimately progress to castration-resistant prostate cancer (CRPC).

Recent advances in next-generation sequencing (NGS) have revealed a variety of molecular characteristics in prostate tumors, including DNA repair deficiencies such as *BRCA* mutations, immune-related alterations, PSMA expression, *SPOP* mutations, and *ERG* fusions (Table 1). Precision medicine based on these findings has already entered clinical practice in some areas, such as with PARP inhibitors and PSMA-targeted therapies. Other approaches, including bipolar androgen therapy (BAT) and treatments targeting *CDK12* mutations, remain under active investigation.

**Figure 1:**
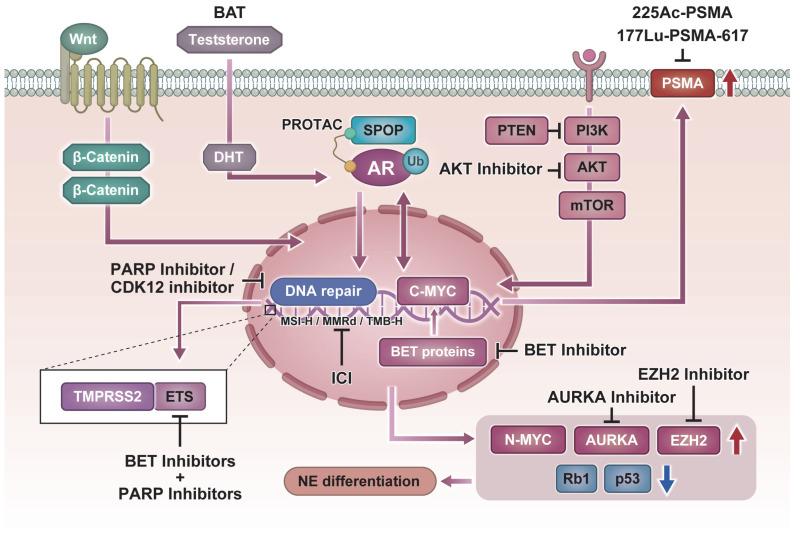
Molecular pathways and precision therapeutic strategies in prostate cancer. Schematic representation of key molecular alterations and their matched targeted therapies.

This review outlines the current state of personalized medicine in prostate cancer, dividing the discussion into clinically implemented treatments and strategies under development, and concludes with perspectives on future directions. An overview of the current landscape of precision medicine in prostate cancer, integrating both established clinical applications and emerging strategies, is summarized in Figure 1.

## 2. Molecularly Targeted Therapies for Prostate Cancer in Clinical Practice

### 2.1. PARP Inhibitors and HRR Gene Alterations as Therapeutic Targets

Approximately 20% of patients with metastatic castration-resistant prostate cancer (mCRPC) harbor mutations in homologous recombination repair (HRR) genes such as *BRCA1/2*, *ATM*, *CHEK2*, and *CDK12* [1]. These tumors exhibit defective DNA damage repair mechanisms, and inhibition of poly (ADP-ribose) polymerase (PARP) leads to accumulation of unrepaired DNA damage, inducing cancer cell–specific “synthetic lethality.” PARP inhibitors leverage this molecular vulnerability, with particularly high efficacy observed in cases with *BRCA1/2* mutations.

In the PROfound trial, olaparib demonstrated significantly prolonged radiographic progression-free survival (rPFS) and overall survival (OS) compared to standard therapy in mCRPC patients harboring *BRCA1/2* or *ATM* mutations, leading to FDA approval in 2020 [2]. Similarly, rucaparib was approved based on results from the TRITON2 trial, which showed efficacy in mCRPC patients with *BRCA* mutations [3]. These approvals have brought gene mutation–based treatment selection into routine clinical practice. It should be noted that while the PROfound trial provides high-level phase III evidence supporting PARP inhibition, the activity observed in several non-BRCA HRR subgroups is derived largely from early-phase or retrospective analyses and remains exploratory [4].

However, the efficacy of PARP inhibitors is limited in non-*BRCA* HRR mutations such as *ATM* or *CDK12*, highlighting the need for novel biomarkers to refine patient selection and for combination strategies that enhance therapeutic sensitivity.

*CDK12* mutations are found in approximately 5–7% of prostate cancers and define a molecular cluster distinct from *BRCA*-associated HRR deficiency. These tumors are characterized by frequent tandem gene fusions, which generate “clustered” neoantigens that may enhance sensitivity to immune checkpoint inhibitors [5].

Combination therapy with talazoparib and enzalutamide has already been approved for patients with mCRPC harboring HRR mutations. Currently, the phase III TALAPRO-3 trial (Primary Completion Date: September 2025) is evaluating this regimen in the mCSPC setting, aiming to expand its indication. In addition, the ongoing EvoPAR-PR01 trial (saruparib, Primary Completion Date: January 2028) will further clarify the role of PARP inhibition in advanced prostate cancer.

Moreover, *CDK12* encodes a kinase that regulates transcriptional elongation by RNA polymerase II; its inactivation selectively silences long genes involved in DNA damage response (DDR) through aberrant polyadenylation [6].

*ATM* mutations, on the other hand, lead to defects in the DDR pathway but do not result in complete HRR loss, limiting the efficacy of PARP inhibition as monotherapy. Preclinical models have shown that *ATM*-deficient tumors are highly dependent on ATR, another DDR kinase, making ATR inhibitors a promising therapeutic option [7].

For non-*BRCA* HRR gene alterations such as *CDK12* and *ATM*, it is critical to develop targeted therapies tailored to each underlying molecular mechanism. For example, combining immune checkpoint inhibitors with PARP inhibitors may be effective for *CDK12*-mutated tumors, while ATR inhibitors may hold promise for those with *ATM* mutations [8,9].

### 2.2. Immune Checkpoint Inhibitors and the Immunophenotype of Prostate Cancer

Prostate cancer is generally considered an “immunologically cold” tumor with limited sensitivity to immune checkpoint inhibitors (ICIs) compared to other malignancies. However, in certain molecular subtypes characterized by mismatch repair deficiency (MMRd), microsatellite instability-high (MSI-H), or high tumor mutational burden (TMB-H), immune activation is enhanced, leading to increased responsiveness to ICIs [10].

Although the prevalence of MMRd/MSI-H/TMB-H in metastatic castration-resistant prostate cancer (mCRPC) is relatively low—estimated at approximately 3–5% [11]—an increasing number of case reports have documented favorable responses to ICIs in these subgroups. Based on such data, the U.S. FDA approved pembrolizumab, an anti-PD-1 antibody, for all solid tumors harboring these biomarkers regardless of tissue origin [12]. Particularly in MSI-H cases, durable tumor shrinkage and prolonged progression-free survival (PFS) have been reported, designating this subgroup as responsive to ICIs.

Recent large-scale studies have shown that MSI-H/dMMR prostate cancers exhibit higher TMB, increased insertion/deletion (indel) counts, and greater neoantigen load compared to other subtypes. In a report by Lenis et al., among 3244 prostate cancer samples, approximately 45% of MSI-H/dMMR cases showed a RECIST-defined response, and 65% achieved a ≥50% PSA reduction (PSA50 response). In contrast, tumors that were TMB-H but microsatellite stable (MSS) had 0% RECIST response and only a 50% PSA50 response [13]. These results suggest that MSI-H/dMMR status may be more essential than TMB alone in determining immune responsiveness.

In a retrospective study by Graham et al., 8 of 17 mCRPC patients (47%) with MMRd/MSI-H who were treated with pembrolizumab achieved a PSA50 response. Notably, 87.5% of these responders maintained treatment without progression for over 12 months [14]. This study also noted that MMRd prostate cancers tend to present with higher Gleason scores and more advanced disease stages, underscoring their distinct clinical and molecular identity.

A subsequent review by the same group emphasized that, although MMRd occurs in only 3–5% of CRPC cases, it represents a highly immunoresponsive subgroup and stands as a success story of precision oncology. The tissue-agnostic FDA approval of pembrolizumab for MMRd tumors illustrates the paradigm shift toward treatment strategies guided by molecular profiling rather than histology [15].

However, common predictive markers of ICI efficacy—such as PD-L1 expression, tumor-infiltrating lymphocyte (TIL) density, and immune gene expression—are generally low in prostate cancer [16], limiting the accuracy of response prediction. Other mutations associated with increased TMB, such as *CDK12* and *POLE/POLD1*, may also contribute to immune sensitivity [5], and comprehensive biomarker panels incorporating these factors are needed.

Additionally, since ICI monotherapy still demonstrates modest response rates in prostate cancer, research efforts are exploring combination strategies, including ICIs with PARP inhibitors or radiation therapy, aimed at enhancing tumor immunogenicity [17]. Such approaches continue to expand the scope of immuno-oncology in prostate cancer treatment.

Various clinical trials have been conducted to evaluate immune checkpoint inhibitors (ICIs), but monotherapy has generally shown limited efficacy in prostate cancer. For example, the completed phase II KEYNOTE-199 trial [18] reported low overall response rates (~3–5%) with pembrolizumab monotherapy, although durable responses were observed in subsets such as MSI-H/dMMR or TMB-high tumors. The subsequent phase III KEYNOTE-641 trial [19] failed to improve OS or rPFS and was terminated for futility. The phase II CheckMate 9KD trial [20] demonstrated clinical activity, particularly with nivolumab plus docetaxel in chemotherapy-naïve mCRPC patients. More recently, the phase II COMBAT trial [21] showed favorable outcomes, including a PSA response rate of 40%, median rPFS of 5.6 months, and median OS of 24.4 months, supporting the safety and potential of BAT plus nivolumab as a combinatorial strategy. Taken together, these findings indicate that while ICIs are not broadly effective as monotherapy, they hold promise in biomarker-selected patients and in combination regimens.

### 2.3. PSMA-Targeted Radioligand Therapy and Advances in Theranostics

Prostate-specific membrane antigen (PSMA) is a transmembrane glycoprotein highly expressed on prostate cancer cells and correlates with tumor aggressiveness and disease progression [22]. Its expression is particularly elevated in metastatic castration-resistant prostate cancer (mCRPC), making it an attractive therapeutic target. PSMA is central to both diagnostic and therapeutic applications—a concept collectively known as theranostics.

For diagnostic purposes, PSMA-targeted PET/CT imaging using ligands such as 68Ga-PSMA-11 and 18F-DCFPyL enables visualization of micrometastases undetectable by conventional imaging, aiding early recurrence detection and treatment planning [23,24]. More recently, new ligands such as 18F-Flotufolastat (rhPSMA-7.3) have been developed, showing promise for improved pelvic imaging due to low background activity in the bladder [25].

Therapeutically, 177Lu-PSMA-617—a radioligand combining the β-emitting isotope 177Lu with a PSMA-targeting moiety—has demonstrated efficacy in selectively delivering radiation to PSMA-positive mCRPC cells. In the VISION trial (NEJM 2021), patients receiving 177Lu-PSMA-617 in addition to standard of care had significantly improved overall survival (OS) and radiographic progression-free survival (rPFS), leading to FDA approval in 2022 [26]. Similarly, in the phase II TheraP trial, the PSA50 response rate was significantly higher in the 177Lu-PSMA-617 group compared to cabazitaxel (66% vs. 37%) [23], also validating the utility of PSMA PET for patient selection.

Additional 177Lu-based compounds, including 177Lu-PSMA-I&T and antibody-conjugated radioligands like 177Lu-J591 and its modified form TLX591, are under investigation in multiple phase II/III trials. In the SPLASH trial, 177Lu-PSMA-I&T demonstrated a median rPFS of 9.5 months [25]. TLX591 has shown favorable tolerability aside from bone marrow toxicity, and its combination with antibody–drug conjugates (ADCs) is gaining interest [25]. Recent reviews have also summarized the development and clinical applications of PSMA-targeted radioligand therapy, highlighting advances in radiopharmaceutical diversity and theranostic strategies [27,28]

Research into alpha-emitting agents, such as 225Ac-PSMA-617, is also advancing. These agents offer stronger cytotoxic effects and are particularly promising in small-volume disease or 177Lu-resistant cases. In a study by Kratochwil et al., the PSA50 response rate with 225Ac-PSMA-617 reached up to 87% [29]. Similarly, 225Ac-J591 yielded positive outcomes, with a phase I trial by Tagawa et al. reporting a PSA50 response rate of 41% [30]. Though side effects such as xerostomia (due to salivary gland uptake) and bone marrow suppression have been observed, the treatments are generally well tolerated.

In comparative terms, 177Lu-PSMA-617 achieved a median OS of 15.3 months and rPFS of 7–8 months in the VISION trial. For 177Lu-PSMA-I&T, median OS has been reported between 10.7 and 27.1 months. Meanwhile, 225Ac-based therapies have demonstrated a median rPFS of approximately 15 months and median OS of 17–18 months, suggesting their potential as future first-line options [31].

A number of clinical trials investigating PSMA-targeted radioligand therapy are currently ongoing. The phase III PSMAfore trial has already supported the regulatory approval of 177Lu-PSMA-617 in patients with mCRPC progressing after ARSI therapy. To further expand its application, the ongoing PSMAddition trial (Primary Completion Date: July 2025) and PSMA-DC trial (Primary Completion Date: December 2027) are evaluating earlier use of 177Lu-PSMA-617 in the mCSPC setting. Moreover, the ActFirst trial (Primary Completion Date: December 2027) is exploring the potential of 225Ac-PSMA, an alpha-emitting radioligand, as a next-generation therapeutic option.

Moving forward, combination therapies—such as with AR inhibitors or PARP inhibitors—are under investigation to enhance efficacy. Addressing patients with low or heterogeneous PSMA expression remains a clinical challenge [32,33]. Optimal patient selection using PSMA PET imaging before therapy and adaptive sequencing based on post-treatment molecular changes will be crucial [25]. Trials such as UpFrontPSMA and LuTectomy are exploring earlier use of PSMA-targeted therapy in neoadjuvant or first-line settings, signaling continued clinical expansion of this strategy [34]. Direct comparison across PSMA-targeted trials is limited by differences in patient selection, particularly the use of PSMA PET–based eligibility versus conventional imaging criteria. In addition to genomic and transcriptional markers of neuroendocrine differentiation, recent evidence highlights the usefulness of somatostatin receptor–targeted imaging such as 68Ga-DOTATATE PET in detecting treatment-emergent neuroendocrine transformation. Analyses from the 3TMPO cohort showed that a subset of patients with discordant 18F-FDG–positive/PSMA-negative lesions also demonstrated uptake on 68Ga-DOTATATE PET, indicating a neuroendocrine phenotype associated with rapid progression and poor prognosis. By revealing disease biology that is often missed by PSMA imaging alone, SSTR-targeted PET may improve risk stratification and also help identify patients who could potentially benefit from emerging somatostatin receptor–directed radioligand approaches in NEPC [35,36].

Additionally, our research has demonstrated that PSMA is not limited to prostate cancer but is also selectively expressed on tumor vasculature in several malignancies including renal cell carcinoma, pancreatic cancer, and hepatocellular carcinoma [37,38]. We have shown that PSMA expression in these tumors promotes angiogenesis. Based on these findings, cross-cancer anti-angiogenic strategies targeting vascular PSMA are under development, potentially broadening the clinical impact of PSMA-targeted therapies in the future.

Several targeted therapies and radioligand therapies are currently being investigated in phase II/III clinical trials (Table 2).

### 2.4. Therapeutic Strategies Targeting PTEN Loss and the PI3K/AKT Pathway

The PI3K/AKT/mTOR signaling pathway plays a critical role in regulating cell growth and survival and represents one of the two major oncogenic pathways in prostate cancer alongside the androgen receptor (AR) pathway. In particular, deletion or inactivation of the tumor suppressor gene *PTEN* leads to constitutive activation of this pathway and is observed in approximately 40–50% of metastatic castration-resistant prostate cancer (mCRPC) cases [39]. AKT activation resulting from *PTEN* loss functions as an AR-independent survival mechanism in tumor cells and contributes to resistance to AR-targeted therapies [40]. Accordingly, therapeutic inhibition of AKT is emerging as a promising strategy in *PTEN*-deficient tumors.

The phase III CAPItello-281 trial (NCT04493853) demonstrated that the AKT inhibitor capivasertib in combination with abiraterone significantly prolonged radiographic progression-free survival (rPFS) in mCRPC patients with *PTEN* loss, with a trend toward improved overall survival (OS). This trial is also evaluating capivasertib plus abiraterone and ADT in patients with *PTEN*-deficient de novo mCSPC, aiming to define its role in this earlier disease setting. By contrast, the parallel CAPItello-280 trial (NCT04493863), which assessed capivasertib with docetaxel in mCRPC, was discontinued after interim analysis due to lack of efficacy. Collectively, these studies support the clinical feasibility of targeting the PI3K/AKT pathway in *PTEN*-altered prostate cancer, while highlighting the importance of biomarker-driven patient selection. While these findings are encouraging, it is important to note that the clinical evidence supporting AKT inhibition remains heterogeneous, with efficacy varying across molecular subgroups and most data outside of CAPItello-281 still derived from early-phase or population-restricted studies.

Recent basic and translational studies have further elucidated how functional aberrations in the PI3K/AKT/mTOR (PAM) pathway—including *PTEN* loss, *PIK3CA/CB* mutations, and AKT activation—drive prostate cancer progression and therapy resistance. As highlighted in a review by Choudhury et al., monotherapy targeting this pathway has shown limited efficacy; however, combination strategies involving taxane-based chemotherapy, PARP inhibitors, or immune checkpoint inhibitors (ICIs) hold promise. The importance of biomarker-driven patient selection in such combination approaches is increasingly emphasized [41].

Glaviano et al. have identified the PAM axis as the most frequently activated oncogenic pathway in human cancers and a key contributor to therapeutic resistance. A variety of inhibitors are under development, including pan-PI3K inhibitors, isoform-specific PI3K inhibitors, ATP-competitive and allosteric mTOR inhibitors, and bi-steric mTOR inhibitors. Strategies involving combination with immunotherapy are also under investigation [42].

Furthermore, Mao et al. demonstrated that *PTEN*-deficient tumors are not universally dependent on p110β (*PIK3CB*). Isoform dependency varies according to the mutational status of *PIK3CA/CB* and the presence of feedback inhibition. Their study showed that AKT inhibitors may overcome IGF1R-mediated resistance more effectively than pan-PI3K inhibitors in *PTEN*-deficient tumors, making AKT inhibition potentially more advantageous in this setting. Conversely, in tumors with intact *PTEN* and activating *PIK3CA* mutations, selective inhibition of p110α was found to be more effective. These findings underscore the critical importance of tailoring therapeutic strategies to the tumor’s molecular profile [43].

Adverse events such as hyperglycemia, rash, and diarrhea have been reported but are generally manageable, suggesting a favorable safety profile for these molecularly targeted therapies. Key areas of future research include direct comparisons between PI3K and mTOR inhibitors, optimization of combinations and sequencing with AR-targeted therapies, and the development of non-invasive diagnostic tools such as cfDNA-based detection of *PTEN* loss. Targeting the PI3K/AKT pathway holds promise as a precision medicine approach, particularly for the large subset of prostate cancer patients harboring *PTEN* loss.

## 3. Novel Therapeutic Strategies and Exploratory Approaches Based on Molecular Subtypes

Several emerging therapeutic strategies for prostate cancer are currently being evaluated in ongoing phase II/III clinical trials, which are summarized in Table 2.

### 3.1. Bipolar Androgen Therapy (BAT) and the Paradoxical Modulation of AR Signaling

Bipolar androgen therapy (BAT) is a novel therapeutic concept that contrasts with conventional androgen deprivation by administering supraphysiological doses of testosterone cyclically. This approach exploits the vulnerability of castration-resistant prostate cancer (CRPC) cells—which are highly dependent on androgen receptor (AR) signaling—to abrupt hormonal fluctuations, thereby inducing cell-cycle arrest and apoptosis [44].

One of the most intriguing aspects of BAT is the observation that tumors resistant to AR-targeted agents can regain sensitivity to these drugs following BAT—a phenomenon referred to as “resensitization” [45]. This strategy leverages adaptive changes in tumor cells by alternating or interrupting therapies, potentially enabling new treatment sequences that combine existing agents with BAT.

Regarding the mechanisms underlying this resensitization, Sena et al. [46] demonstrated in various preclinical models and patient samples that BAT’s antitumor effects rely on high AR activity. Tumors with elevated AR activity showed significant suppression of MYC expression, contributing to tumor regression. Furthermore, BAT downregulates AR itself, which may lead to acquired resistance. However, alternating BAT with the AR inhibitor enzalutamide reinduces AR expression, re-sensitizing tumors to BAT. This dynamic modulation suggests a potential strategy for personalized BAT regimens based on AR activity biomarkers and highlights the feasibility of cyclic treatments combining AR blockade and activation [47].

Clinically, BAT’s distinct therapeutic and resensitization effects are under evaluation. In cohort C of the RESTORE trial (a phase II, single-center, multi-cohort study), 29 treatment-naïve CRPC patients received BAT (testosterone cypionate 400 mg every four weeks). Although only 14% achieved a PSA50 response during BAT, an impressive 94% of the 18 patients subsequently rechallenged with enzalutamide or abiraterone achieved a PSA50 response, and 83% achieved a PSA90 response [46]. These findings support BAT’s role not only as a direct antitumor agent but also as a therapeutic “primer” that can restore tumor sensitivity to AR-targeted therapies. Median radiographic progression-free survival (rPFS) with BAT alone was 8.5 months; however, PFS after rechallenge with AR inhibitors had not been reached at a median follow-up of 26.4 months, indicating durable benefit. Although BAT has demonstrated biologically intriguing responses, current evidence is primarily derived from small, single-center phase II studies and should therefore be interpreted as preliminary.

In addition, BAT may offer advantages in terms of quality of life (QOL). In the TRANSFORMER trial, patients receiving BAT reported trends toward improved QOL compared to those on enzalutamide alone [44]. Moreover, combination strategies involving BAT and PARP inhibitors (e.g., olaparib) are under investigation, as BAT has been shown to transiently induce DNA damage, potentially enhancing the synthetic lethality of PARP inhibition [47].

Nonetheless, BAT has not yet been established as a standard of care. Further studies are required to determine optimal dosing schedules, long-term efficacy, and safety. Since AR dependence plays a central role in treatment response, patient selection remains a key challenge moving forward.

Overall, BAT represents a promising novel hormonal manipulation strategy for CRPC, and its further clinical development is highly anticipated.

### 3.2. CDK12 Mutations and Sensitivity to Immunotherapy

*CDK12* (cyclin-dependent kinase 12) mutations represent a relatively rare genomic alteration observed in approximately 5–7% of prostate cancers. *CDK12* plays a crucial role in regulating the expression of genes involved in DNA damage response (DDR) and transcriptional elongation. Its inactivation is characterized by the expression of large clusters of neoantigens, termed “neoantigen clusters.”

These neoantigen clusters may contribute to enhanced tumor immunogenicity, and tumors harboring *CDK12* mutations are thought to exhibit increased sensitivity to immune checkpoint inhibitors (ICIs). In fact, retrospective analyses have reported clinical responses to ICIs such as pembrolizumab in a subset of prostate cancer patients with *CDK12* mutations [48,49].

Additionally, a comprehensive genomic analysis by Wu et al. revealed that metastatic castration-resistant prostate cancer (mCRPC) with biallelic inactivation of *CDK12* constitutes an immunogenic subtype distinct from ETS fusions, *SPOP* mutations, and homologous recombination deficiency (HRD). These tumors are marked by focal tandem duplications (FTDs), which lead to the formation of fusion genes and a higher burden of chimeric neoantigens. Increased T-cell infiltration and clonal expansion within the tumor further support the immunogenic nature of this subtype, suggesting compatibility with ICI-based therapy [5].

In a detailed immune profiling study, Lotan et al. reported a significant enrichment of CD4^+^FOXP3^+^ immunosuppressive T cells in *CDK12*-mutated tumors. While neoantigen-driven immune activation was evident, the coexistence of immunosuppressive elements in the tumor microenvironment explains why not all *CDK12*-altered cases respond to ICI treatment [50].

Clinically, the efficacy of ICIs in *CDK12*-mutant prostate cancer has shown promise, albeit limited. In a multicenter retrospective study by Antonarakis et al., approximately 33.3% of *CDK12*-mutated patients treated with PD-1 inhibitors (pembrolizumab or nivolumab) demonstrated a PSA response. In contrast, the same cohort showed poor responses to PARP inhibitors and taxane-based chemotherapy, underscoring the relative resistance to standard therapies and the potential responsiveness to ICIs [49].

Moreover, *CDK12*-deficient tumors frequently exhibit an “immune-activated” transcriptional profile, including increased tumor-infiltrating lymphocytes (TILs) and elevated interferon signatures [49,50]. These features likely underpin the observed immunotherapy sensitivity in this molecular subtype.

However, clinical responses remain heterogeneous, and not all patients with *CDK12* mutations benefit from ICI treatment. Therefore, whether *CDK12* status alone is a sufficient predictive biomarker is still under debate. Additional immune-related markers such as tumor mutational burden (TMB), PD-L1 expression, and TIL density are being explored as complementary predictors of response.

Ongoing phase 1 and 2 clinical trials (e.g., NCT03572478) are currently evaluating the efficacy of ICI therapy in *CDK12*-mutant prostate cancer. As evidence continues to accumulate, *CDK12* mutation status may become increasingly important in shaping future immunotherapy strategies.

### 3.3. Targeting SPOP Mutations and Protein Degradation Mechanisms/ERG Fusions and Transcriptional Regulation

In prostate cancer, mutations in *SPOP* (Speckle-type POZ protein) and TMPRSS2–*ERG* gene fusions are recognized as distinct molecular subtypes. *SPOP* mutations are primarily observed in treatment-naïve hormone-sensitive prostate cancer, occurring in approximately 10–15% of cases, though their frequency tends to decrease in metastatic or castration-resistant disease (CRPC). In contrast, *ERG* fusions, resulting from structural rearrangements, are detected in nearly half of prostate cancers and are particularly common in tumors with strong androgen dependency.

*SPOP* mutations disrupt its function as an adaptor protein within the E3 ubiquitin ligase complex, impairing the degradation of AR coactivators such as SRC-3 and BRD4, as well as BET family proteins. This leads to excessive activation of AR signaling. Consequently, *SPOP*-mutant tumors may exhibit heightened sensitivity to AR-targeted therapies such as enzalutamide and abiraterone [51]. Furthermore, clinical trials using BET inhibitors, such as ZEN-3694, which target epigenetic transcription regulators like BRD4, are underway and have shown promising combinatorial effects [52].

A noteworthy development is the PROTAC (Proteolysis-Targeting Chimera) technology. This next-generation pharmacological strategy involves directing E3 ligases to target proteins (e.g., AR, BRD4), promoting their ubiquitination and proteasomal degradation. Unlike traditional inhibitors, PROTACs aim to eliminate the protein entirely [53].

On the other hand, *ERG* fusions arise from androgen-induced overexpression of the fusion gene between TMPRSS2 and the transcription factor *ERG*. *ERG* activates transcriptional programs related to cell invasion, metastasis, and angiogenesis. However, due to its nature as a transcription factor, direct targeting is difficult. Indirect inhibition via BRD4 and PARP1—proteins that functionally interact with *ERG*—is being explored, and preclinical studies suggest that combined use of BET and PARP inhibitors may be a promising strategy [54].

Importantly, *SPOP* mutations and *ERG* fusions are mutually exclusive and rarely co-occur. Each represents a distinct molecular cluster and tumor progression pathway, emphasizing the need for personalized therapeutic approaches based on individual molecular profiles.

Additionally, we have previously reported that *SPOP* is involved in DNA repair via TOP2A. *SPOP* mutations may disrupt this mechanism, potentially sensitizing tumors to TOP2A inhibitors or PARP inhibitors. This opens new avenues for exploiting DNA damage response pathways as therapeutic targets in *SPOP*-mutant tumors [55,56].

Further investigations have revealed the functional antagonism between *SPOP* and *ERG*. Wild-type *SPOP* promotes proteasomal degradation of *ERG* by recognizing an N-terminal degron motif. However, this degradation is impeded when *SPOP* is mutated or when *ERG* proteins are truncated due to TMPRSS2–*ERG* fusion events that eliminate the degron motif. As a result, *ERG* proteins are aberrantly stabilized, contributing to increased tumor aggressiveness and metastasis. Thus, both *SPOP* mutations and *ERG* fusions drive tumor progression through distinct but converging mechanisms [57,58].

Bernasocchi et al. provided mechanistic insight into the mutual exclusivity of *SPOP* and *ERG* alterations. In *ERG*-positive tumors with wild-type *SPOP*, degradation of ZMYND11 maintains *ERG* activity while suppressing AR signaling. In contrast, in *SPOP*-mutant tumors, ZMYND11 is stabilized, repressing *ERG* activity and enhancing AR signaling. This antagonistic relationship influences therapeutic responses: *SPOP*-mutant tumors tend to respond to androgen deprivation therapy, whereas *ERG*-positive tumors may be more sensitive to high-dose androgen therapy or *SPOP* inhibitors [59].

Thus, both *SPOP* mutations and *ERG* fusions regulate *ERG* stability and AR activity through divergent pathways. Treatment selection based on the underlying molecular context is expected to play an increasingly important role in clinical decision-making.

### 3.4. Approaches to Neuroendocrine Prostate Cancer (NEPC)

Neuroendocrine prostate cancer (NEPC) is a highly treatment-resistant and aggressive subtype of prostate cancer that typically emerges after prolonged suppression of the androgen receptor (AR) pathway through androgen deprivation therapy (ADT) or next-generation AR inhibitors. Morphologically, NEPC resembles small cell or large cell neuroendocrine carcinomas. Clinically, it often presents with rapid visceral metastases to organs such as the liver or bones, and is typically associated with low or undetectable PSA levels [60].

At the molecular level, NEPC is characterized by a shift away from AR dependence, with frequent alterations including N-Myc overexpression, Aurora kinase A (AURKA) activation, and loss or inactivation of *TP53* and *RB1*. Based on these changes, therapeutic strategies targeting transcriptional regulation, such as the use of Aurora kinase A inhibitors (e.g., alisertib) and EZH2 inhibitors, have been explored. Preclinical models have demonstrated that these agents can suppress tumor cells exhibiting NEPC-like phenotypes [61].

Additionally, the tumor microenvironment in NEPC is highly immunosuppressive, limiting the efficacy of immune checkpoint inhibitors (ICIs) when used as monotherapy. Our spatial transcriptomic analysis has revealed that de novo NEPC exhibits profoundly low T cell infiltration compared to AR-positive prostate cancer (ARPC) and is marked by strong activation of immunosuppressive pathways including TGF-β and IL-10 signaling. These features may underlie the limited clinical benefit of ICIs in NEPC [62]. Nevertheless, combination strategies involving ICIs and EZH2 or AURKA inhibitors may enhance immune responses, representing a promising area of future investigation.

The lack of standardized treatments and atypical clinical presentation of NEPC often leads to delayed diagnosis. Early detection strategies, such as molecular profiling through cfDNA and liquid biopsy, and the identification of predictive biomarkers for NEPC transformation, are urgently needed. Furthermore, “preventive” interventions to suppress lineage plasticity—the transition from AR-driven adenocarcinoma to NEPC—are currently under exploration [63].

In addition to genomic and transcriptional markers of neuroendocrine differentiation, emerging evidence highlights the value of somatostatin receptor–targeted PET imaging, such as 68Ga-DOTATATE PET, in detecting treatment-emergent NEPC and identifying lesions that have undergone a neuroendocrine “switch.” This approach has attracted increasing interest from a theranostic perspective, as SSTR-positive disease may represent a potential candidate population for future somatostatin receptor–targeted radioligand therapies (e.g., 177Lu-DOTATATE), which are now being explored in aggressive-variant prostate cancer and NEPC-like phenotypes. Recent analyses from the 3TMPO cohort demonstrated that a subset of patients with discordant 18F-FDG–positive/PSMA-negative lesions also exhibited uptake on 68Ga-DOTATATE PET, suggesting neuroendocrine transformation and identifying patients at particularly high risk for rapid progression and poor prognosis. These findings indicate that SSTR-targeted PET may complement PSMA imaging by revealing neuroendocrine phenotypes that are otherwise missed, thereby refining risk stratification and improving selection of candidates for emerging theranostic strategies in NEPC [35,36].

Of particular interest is the role of N-Myc overexpression in driving epigenetic reprogramming in NEPC. N-Myc cooperates with AR co-factors like *FOXA1* and *HOXB13* to activate transcriptional programs associated with neural lineage commitment, thereby promoting an AR-independent neuroendocrine phenotype. EZH2 inhibitors have been shown to partially reverse this N-Myc–driven reprogramming, offering a potential therapeutic strategy [64,65].

The selective EZH2 inhibitor mevrometostat (PF-06821497) is currently being evaluated in the phase II MEVPRO-1 trial (NCT04179864) and the phase III MEVPRO-2 trial (NCT05934220), both of which are designed to assess efficacy in aggressive-variant or NEPC-like prostate cancers. Early-phase studies suggest that EZH2 inhibition may reverse lineage plasticity and restore sensitivity to androgen receptor signaling inhibitors. Results from these ongoing trials will clarify the therapeutic potential of EZH2 inhibition as part of future strategies against NEPC. Given that most studies in NEPC are early-phase and include heterogeneous patient populations, current evidence should be interpreted with caution until validated in larger prospective trials.

Molecular studies have revealed that the emergence of NEPC results from transcriptional dysregulation triggered by inactivation of *TP53* and *RB1*, EZH2 overexpression, and activation of neural lineage-specifying transcription factors such as *SOX2*, *ASCL1*, and *BRN2*. This phenomenon, known as “lineage plasticity,” reflects the ability of tumor cells to transition from AR-dependent adenocarcinoma to a neuroendocrine state, thereby conferring resistance to AR-targeted therapies [66,67].

Furthermore, approximately 15–20% of treatment-resistant prostate cancers exhibit loss of AR expression and signaling, accompanied by the appearance of neural markers and stem-like phenotypes. This process can also include transdifferentiation to a “double-negative” subtype (negative for both AR and neuroendocrine markers). Defining the pathological features and establishing therapeutic targets for these evolving tumor states remains a critical clinical challenge [68].

## 4. Conclusions and Future Perspectives

Precision medicine in prostate cancer is undergoing a significant paradigm shift, driven by recent advances in genomic profiling and biomarker research. Therapeutic strategies such as PARP inhibitors, immune checkpoint inhibitors, PSMA-targeted radioligand therapy, and AKT inhibitors have already begun clinical implementation, making molecularly guided treatment selection a practical reality.

Meanwhile, emerging strategies—including those targeting *SPOP* mutations, *CDK12* alterations, *ERG* fusions, bipolar androgen therapy (BAT), and neuroendocrine prostate cancer (NEPC)—have yet to become standard of care, but accumulating evidence suggests their potential efficacy in selected molecular subgroups. Optimizing treatment approaches for these subtypes and refining biomarker-driven patient selection will be essential moving forward.

In addition, the advancement of liquid biopsy technologies such as cell-free DNA (cfDNA), the integration of imaging and genomic data through artificial intelligence, and the application of multi-omics analyses are expected to further enable the realization of truly personalized medicine. Moreover, radiomics and radiogenomics have emerged as promising approaches to noninvasively characterize tumor biology by linking quantitative imaging features with genomic alterations. Recent work has shown that PSMA PET uptake patterns, MRI-derived radiomic signatures, and metabolic phenotypes on FDG or DOTATATE PET can reflect key molecular events such as PTEN loss, DNA repair deficiencies, or neuroendocrine differentiation [35]. As these imaging–genomic associations become better defined, radiogenomic tools may help refine prognostic assessment and guide the choice between systemic therapies and radioligand-based approaches in advanced prostate cancer. Recent reviews further emphasize the importance of integrating radiomics, genomics, and biomarker-driven approaches to refine individualized risk stratification in prostate cancer [69].

The expansion of precision oncology will not merely involve the introduction of new therapies but has the potential to fundamentally reshape the entire clinical paradigm of prostate cancer management.

## Figures and Tables

**Table 1:** Precision Oncology Map in Prostate Cancer. Summary of key genetic alterations and matched targeted therapies.

Genetic Alteration	Matched Therapy
HRR mutations (e.g., *BRCA1/2*)	PARP inhibitors (*Olaparib*, *Rucaparib*)
MSI-H/MMRd	Immune checkpoint inhibitors (*Pembrolizumab*)
PTEN loss	AKT inhibitors (*Capivasertib*)
PSMA-positive	Radioligand therapy (177Lu-PSMA-617)
SPOP mutations	AR pathway inhibitors, BET inhibitors, or PROTACs (targeting AR or BRD4)
NEPC phenotype	AURKA inhibitors (*Alisertib*), EZH2 inhibitors

**Table 2:** Ongoing Clinical Trials in Prostate Cancer. Representative ongoing phase II/III clinical trials investigating targeted therapies, radioligand therapy, and immunotherapy in prostate cancer.

Target	Trial Name	Intervention	Population	Phase	PCD	NCT No.
PARP	TALAPRO-3	Talazoparib + Enzalutamide	mCSPC, HRR mutated	III	September 2025	NCT04821622
	EvoPAR-PR01	Saruparib + NHA	mCSPC, HRR and non-HRR	III	January 2028	NCT06120491
PSMA	PSMAddition	^177^Lu-PSMA-617 + ARSI + ADT	mCSPC, PSMA+	III	July 2025	NCT04720157
	PSMA-DC	^177^Lu-PSMA-617 ± Docetaxel	mCSPC, PSMA+	III	December 2027	NCT05939414
	ActFirst	^225^Ac-PSMA-617	mCSPC, PSMA+	III	December 2027	NCT06231234
EZH2	MEVPRO-1	Mevrometostat (PF-06821497)	NEPC/aggressive variant	II	Ongoing	NCT04179864
	MEVPRO-2	Mevrometostat + SOC	NEPC-like prostate cancer	III	Ongoing	NCT05934220

## Data Availability

No new data were created or analyzed in this study. Data sharing is not applicable to this article.

## Abbreviations

The following abbreviations are used in this manuscript:

PCa	Prostate cancer
CRPC	Castration-resistant prostate cancer
ADT	Androgen deprivation therapy
AR	Androgen receptor
NGS	Next-generation sequencing
HRR	Homologous recombination repair
BRCA	Breast cancer gene (BRCA1/2)
ATM	Ataxia telangiectasia mutated
CHEK2	Checkpoint kinase 2
CDK12	Cyclin-dependent kinase 12
PARP	Poly (ADP-ribose) polymerase
MSI-H	Microsatellite instability-high
MMRd	Mismatch repair deficiency
TMB	Tumor mutational burden
ICI	Immune checkpoint inhibitor
PSMA	Prostate-specific membrane antigen
rPFS	Radiographic progression-free survival
OS	Overall survival
PET	Positron emission tomography
ADC	Antibody–drug conjugate
PI3K	Phosphoinositide 3-kinase
AKT	Protein kinase B
mTOR	Mechanistic target of rapamycin
PTEN	Phosphatase and tensin homolog
BAT	Bipolar androgen therapy
TIL	Tumor-infiltrating lymphocyte
NEPC	Neuroendocrine prostate cancer
cfDNA	Cell-free DNA
FTD	Focal tandem duplication
PROTAC	Proteolysis-targeting chimera
SPOP	Speckle-type POZ protein
ERG	ETS-related gene
ARSI	Androgen receptor signaling inhibitor
